# Three-year mortality in 30-day survivors of critical care with acute kidney injury: data from the prospective observational FINNAKI study

**DOI:** 10.1186/s13613-016-0218-5

**Published:** 2016-11-29

**Authors:** Henriikka Mildh, Ville Pettilä, Anna-Maija Korhonen, Sari Karlsson, Tero Ala-Kokko, Matti Reinikainen, Suvi T. Vaara, the FINNAKI Study Group, Raili Laru-Sompa, Anni Pulkkinen, Minna Saarelainen, Mikko Reilama, Sinikka Tolmunen, Ulla Rantalainen, Marja Miettinen, Markku Suvela, Katrine Pesola, Pekka Saastamoinen, Sirpa Kauppinen, Ville Pettilä, Kirsi-Maija Kaukonen, Sara Nisula, Suvi Vaara, Raili Suojaranta-Ylinen, Leena Mildh, Mikko Haapio, Laura Nurminen, Sari Sutinen, Leena Pettilä, Helinä Laitinen, Heidi Syrjä, Kirsi Henttonen, Elina Lappi, Hillevi Boman, Tero Varpula, Päivi Porkka, Mirka Sivula, Mira Rahkonen, Anne Tsurkka, Taina Nieminen, Niina Pirttinen, Ari Alaspää, Ville Salanto, Hanna Juntunen, Teija Sanisalo, Ilkka Parviainen, Ari Uusaro, Esko Ruokonen, Stepani Bendel, Niina Rissanen, Maarit Lång, Sari Rahikainen, Saija Rissanen, Merja Ahonen, Elina Halonen, Eija Vaskelainen, Meri Poukkanen, Esa Lintula, Sirpa Suominen, Jorma Heikkinen, Timo Lavander, Kirsi Heinonen, Anne-Mari Juopperi, Tadeusz Kaminski, Fiia Gäddnäs, Tuija Kuusela, Jane Roiko, Sari Karlsson, Matti Reinikainen, Tero Surakka, Helena Jyrkönen, Tanja Eiserbeck, Jaana Kallinen, Tero Ala-Kokko, Jouko Laurila, Sinikka Sälkiö, Vesa Lund, Päivi Tuominen, Pauliina Perkola, Riikka Tuominen, Marika Hietaranta, Satu Johansson, Seppo Hovilehto, Anne Kirsi, Pekka Tiainen, Tuija Myllärinen, Pirjo Leino, Anne Toropainen, Anne Kuitunen, Jyrki Tenhunen, Ilona Leppänen, Markus Levoranta, Sanna Hoppu, Jukka Sauranen, Atte Kukkurainen, Samuli Kortelainen, Simo Varila, Outi Inkinen, Niina Koivuviita, Jutta Kotamäki, Anu Laine, Simo-Pekka Koivisto, Raku Hautamäki, Maria Skinnar

**Affiliations:** 1 Division of Intensive Care Medicine, Department of Anesthesiology, Intensive Care and Pain Medicine, University of Helsinki and Helsinki University Hospital, Helsinki, Finland; 2 Department of Intensive Care Medicine, Inselspital, Bern University Hospital, University of Bern, Bern, Switzerland; 3 Critical Care Medicine Research Group, Tampere University Hospital, Tampere, Finland; 4 Department of Anesthesiology, Division of Intensive Care Medicine, Oulu University Hospital, Medical Research Centre Oulu, University of Oulu, Oulu, Finland; 5 Department of Intensive Care, North Karelia Central Hospital, Joensuu, Finland

**Keywords:** Acute kidney injury, Renal replacement therapy, Long-term mortality, Intensive care

## Abstract

**Background:**

The role of an episode of acute kidney injury (AKI) in long-term mortality among initial survivors of critical illness is controversial. We aimed to determine whether AKI is independently associated with decreased survival at 3 years among 30-day survivors of intensive care.

**Results:**

We included 2336 30-day survivors of intensive care enrolled in the FINNAKI study conducted in seventeen medical–surgical ICUs in Finland during a 5-month period in 2011–2012. The incidence of AKI, defined by the Kidney Disease: Improving Global Outcomes criteria, was 34.6%, and 192 (8.3%) commenced RRT. The 3-year mortality among AKI patients was 23.5% (95% CI 20.6–26.4%) compared to 18.9% (17.0–20.9%) of patients without AKI, *p* = 0.01. However, after adjustments using Cox proportional hazards regression, AKI was not associated with decreased 3-year survival (HR 1.05; CI 95% 0.86–1.27), whereas advanced age, poor pre-morbid functional performance, and presence of several comorbidities were. Additionally, we matched AKI patients to non-AKI patients 1:1 according to age, gender, presence of severe sepsis, and a propensity score to develop AKI. In the well-balanced matched cohort, 3-year mortality among AKI patients was 136 of 662 (20.5%; 17.5–23.6%) and among matched non-AKI patients 143 of 662 (21.6%; 18.5–24.7%), *p* = 0.687. Neither AKI nor RRT was associated with decreased survival at 3 years in the sensitivity analyses that excluded patients (1) with chronic kidney disease, (2) with AKI not commenced renal replacement therapy (RRT), and (3) with estimated pre-admission creatinine, chronic kidney disease, or AKI stage 1.

**Conclusion:**

AKI was not an independent risk factor for 3-year mortality among 30-day survivors. Increased 3-year mortality among patients with AKI who survive critical illness may not be related to AKI per se, but rather to advanced age and pre-existing comorbidities.

**Electronic supplementary material:**

The online version of this article (doi:10.1186/s13613-016-0218-5) contains supplementary material, which is available to authorized users.

## Background

 Acute kidney injury (AKI) is a frequently encountered syndrome in the critically ill, with an incidence varying from 15% up to 40% [1–5]. AKI can markedly increase the severity of acute illness [5, 6] as well as the length of intensive care unit (ICU) and hospital stay [1, 2, 5, 6]. Moreover, patients with AKI are susceptible to the later development of chronic kidney disease (CKD) [4, 7].

AKI has been significantly associated with increased short-term mortality [3, 4, 6, 8], but results about its impact on increased long-term mortality among initial survivors of AKI and critical illness are few and conflicting. A large cohort study revealed the 1-year mortality of 30-day survivors of AKI defined by RIFLE (risk, injury, failure, loss, end-stage renal disease) creatinine (Cr) criteria to range from 20.5 to 23.8% with increasing severity of AKI, compared to 10.7% of the patients without AKI [5]. A 10-year follow-up study among critically ill patients found stage 1 AKI patients to have worse crude survival rate than patients without AKI [9]. The difference was significant also among 28-day survivors, but became nonsignificant after adjusting for confounders [9]. Additionally, 30-day survivors commenced renal replacement therapy (RRT) did not have worse 3-year outcome compared to their propensity-matched control group of hospitalized patients without AKI [10]. Moreover, among all ICU patients, the short-term mortality has been related mostly to the type and severity of acute illness, whereas long-term mortality among initial survivors has been primarily determined by patient age and comorbidities [11], which are also well-known predisposing factors for the development of AKI [1].

Thus, we hypothesized that critically ill patients with AKI have decreased long-term survival compared to patients without AKI, but that pre-existing comorbidities and advanced age could be explanatory factors. Therefore, in this analysis among 30-day survivors of intensive care, we aimed to determine whether AKI is independently associated with increased 3-year mortality.

## Methods

We performed a prospective cohort study in 17 ICUs in Finland between September 1, 2011, and February 1, 2012 [3]. The Ethics Committee of the Department of Surgery at Helsinki University Hospital gave approval for the study protocol and the use of deferred consent with written informed consent obtained from the patient or proxy as soon as possible. The Finnish National Institute of Health and Welfare approved collection of data of deceased patients from medical records if an informed consent could not be obtained.

### Patients

All adult (>18 years) patients with an emergency admission to the study ICUs of any duration, or an elective admission expecting to last over 24 h, were included to this Finnish Acute Kidney Injury (FINNAKI) study [3]. Patients who (1) were on chronic dialysis, (2) were readmitted and had received RRT during their previous admission, (3) were organ donors, (4) had insufficient language skills for giving informed consent or were not permanent Finnish residents, (5) were transferred from another ICU and had already been included for the study data collection period of five days, or (6) who were intermediate care patients were excluded from the study. We followed the 2901 included patients [3] until 3 years. For this current analysis, we excluded all 30-day non-survivors (*N* = 548), those who had previously received any organ transplant (*N* = 16) or had acquired immune deficiency syndrome (AIDS) (*N* = 1). As a further explanatory analysis regarding potential survival bias, we present data from the entire FINNAKI cohort (*n* = 2901) without exclusions.

### Data collection

We prospectively collected data on previous and present medical status, patient demographics, ICU severity scores, presence of sepsis, possible risk factors for AKI, and existing comorbidities with study-specific case report form and from the database of the Finnish Intensive Care Consortium (Tieto Ltd, Helsinki, Finland). Data regarding ICU treatment were collected until day 5. Finnish Population Register Centre provided the survival data and the Finnish Registry for Kidney Diseases data on need for chronic dialysis at 3 years.

### Definitions

 We measured plasma Cr concentration daily and urine output hourly and used the Kidney Disease: Improving Global Outcomes (KDIGO) [1] criteria to define and stage AKI, using both Cr and urine output criteria. As the baseline Cr, we used the latest value from previous year excluding the previous week. If it was unavailable, we estimated it by using the Modification of Diet in Renal Disease (MDRD) equation assuming a glomerular filtration rate (GFR) of 75 ml/min/1.73 m^2^ [12]. End-stage renal disease (ESRD) was defined by need for maintenance dialysis at least for three months [13]. We defined sepsis according to the American College of Chest Physicians/Society of Critical Care Medicine Consensus Conference [14].

### Cox model

We used Cox proportional hazards model to adjust for confounders related to survival at 3 years. We tested the validity of the proportional hazards assumption for Cox using the cox.zph method of the R survival package (hhtp://R-project.org) and found it to be valid in all models. We adjusted for gender, type of admission, pre-morbid functional performance, presence of severe sepsis, or comorbidities (arteriosclerosis, chronic obstructive pulmonary disease (COPD), CKD, diabetes mellitus, hypertension, liver failure, malignancy, rheumatoid diseases, systolic heart failure, and thrombophilia), Simplified Acute Physiology Score (SAPS) II points without age and renal components, and use of vasoactive drugs in addition to the presence of AKI. These covariates were chosen based on their distribution between AKI and non-AKI groups. Patients with missing data were assumed not to have the chronic condition. We also performed three sensitivity analyses by excluding (1) patients with CKD, (2) patients with AKI who did not receive RRT, (3) those with an estimated pre-admission Cr, or pre-existing CKD, or AKI stage 1. As a further explanatory analysis, time-stratified Cox models were generated as suggested [15].

### Matching

As age and comorbidities are risk factors for both AKI [3, 6] and increased long-term mortality [11], we performed a matched analysis between AKI and non-AKI patients. We matched patients 1:1 according to (1) age (caliper width ± 5 years), (2) sex, (3) presence of severe sepsis in ICU, and (4) the logit of propensity score for developing AKI (caliper width 0.2 SD) at random and without replacement. The logistic regression model (presented in Additional file 1: Table S1) used to construct the propensity score for AKI included variables previously reported to be associated with development of AKI [1, 3, 16] and the outcome as recommended [17]. We calculated standardized differences between the matched groups to assess the post-matching balance and considered standardized differences less than 10% indicative of good post-matching balance [18].

### Statistical analysis

We present continuous data as median with interquartile range (IQR) and categorical data as absolute number and percentage. We used Chi-square test for categorical values and Mann–Whitney *U* test for continuous data in comparisons. We considered two-sided *p* value <0.05 as significant. In the matched sample, we compared categorical data with the McNemar test. We calculated the 95% CI for the difference in the 3-year mortality in the matched groups with Newcombe’s method [19]. We used SPSS version 23 (SPSS, Armonk, NY, USA) and R (http://R-project.org) for data analysis.

## Results

### Included patients

**Fig. 1:**
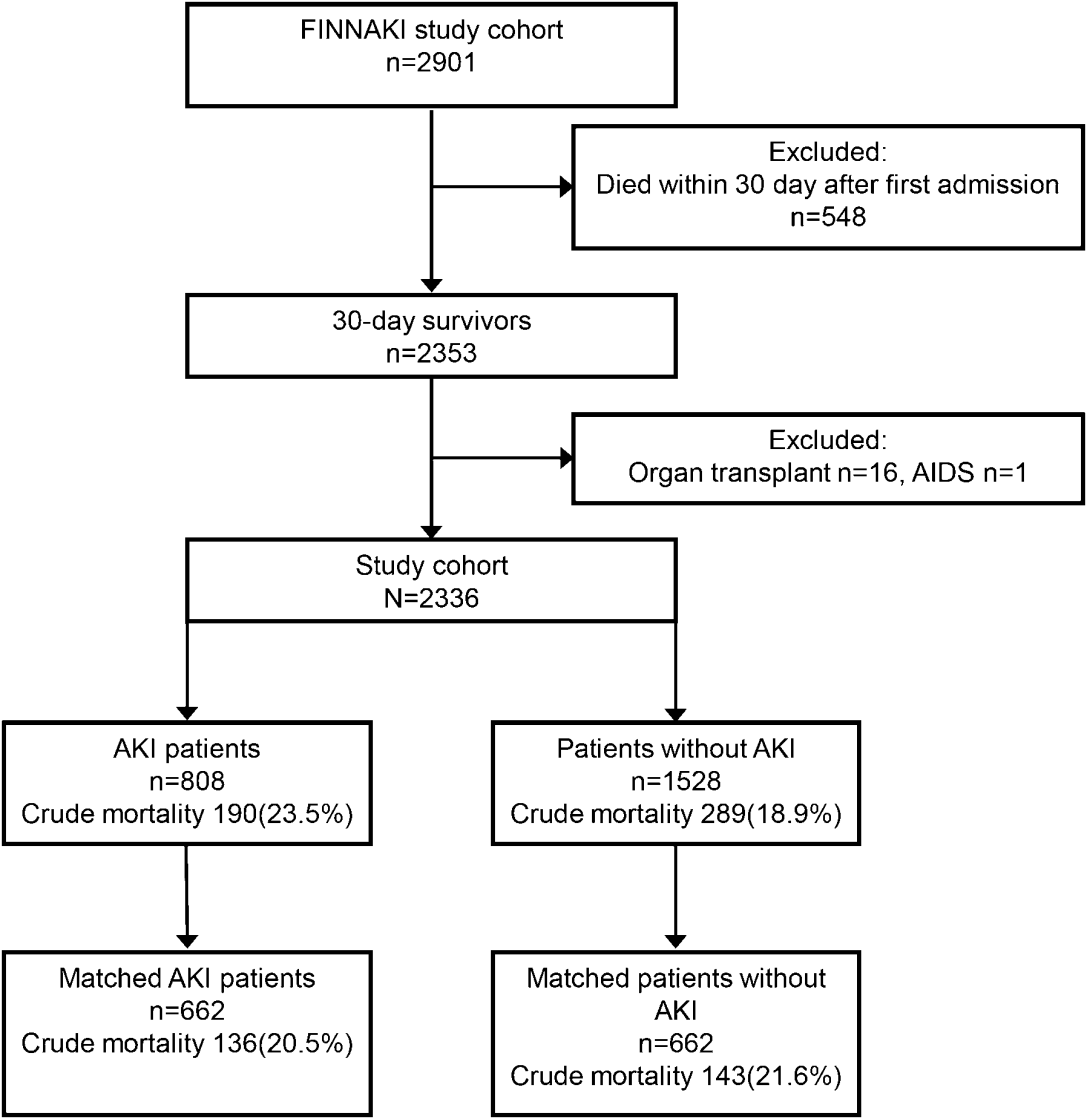
Study flowchart. *AIDS* acquired immune deficiency syndrome, *AKI* acute kidney injury, *FINNAKI* Finnish Acute Kidney Injury

Altogether 2336 30-day survivors were included in the final analysis (study flowchart in Fig. 1). The incidence of AKI was 808/2336 (34.6%; 95% CI 32.7–36.5%), including 378 (16.2%) patients with stage 1, 162 (6.9%) with stage 2, and 268 (11.5%) with stage 3 AKI. During the first five days in ICU, 192 (8.3%; 95% CI 7.1–9.4%) patients commenced RRT. Table 1 presents characteristics of all study patients according to the presence of AKI.

**Table 1:** Characteristics of study patients (*N* = 2336) and comparison between patients with and without acute kidney injury

Characteristics	Data available	All *N* = 2336	No AKI *N* = 1528	AKI *N* = 808	*p* value
Age (years)	2336	62.0 (49.0–72.0)	61.0 (47.0–71.0)	64.0 (53.0–73.8)	<0.001
Gender (male)	2336	1479 (63.3)	951 (61.6)	538 (66.6)	0.019
Operative admission	2336	886 (37.9)	577 (37.8)	309 (38.2)	0.823
Emergency admission	2336	2007 (85.9)	1312 (85.9)	695 (86.0)	0.950
Pre-morbid functional performance	2331				0.002
Normal		1246 (53.5)	858 (56.2)	388 (48.2)	
Disabled to work but no need for assistance		803 (34.4)	500 (32.8)	303 (37.6)	
Some assistance required		216 (9.3)	131 (8.6)	85 (10.6)	
Totally dependent on assistance		66 (2.8)	37 (2.4)	29 (3.6)	
APACHE diagnosis main groups	2336				<0.001
Respiratory		264 (11.3)	178 (11.6)	86 (10.6)	
Metabolic		244 (10.4)	181 (11.8)	63 (7.8)	
Neurological		110 (4.7)	100 (6.5)	10 (1.2)	
Gastrointestinal, non-op		139 (6.0)	81 (5.3)	58 (7.2)	
Gastrointestinal, post-op		204 (8.7)	119 (7.8)	85 (10.5)	
Sepsis		126 (5.4)	61 (4.0)	65 (8.0)	
Cardiovascular, non-op		244 (10.4)	155 (10.1)	89 (11.0)	
Cardiovascular, post-op		400 (17.1)	240 (15.7)	160 (19.8)	
Trauma, non-op		118 (5.1)	92 (6.0)	26 (3.2)	
Arteriosclerosis	2336	279 (11.9)	158 (10.3)	121 (15.0)	0.001
Chronic kidney disease	2336	134 (5.7)	53 (3.5)	81 (10.0)	<0.001
Chronic obstructive pulmonary disease	2336	203 (8.7)	127 (8.3)	76 (9.4)	0.396
Diabetes mellitus	2332	507 (21.7)	297 (19.5)	210 (26.0)	<0.001
Hypertension	2336	1072 (45.9)	630 (41.2)	442 (54.7)	<0.001
Chronic liver failure	2336	70 (3,0)	43 (2.8)	27 (3.3)	0.524
Malignancy	2200	63 (2.9)	44 (3.0)	19 (2.5)	0.504
Rheumatoid disease	2336	100 (4.3)	63 (4.1)	37 (4.6)	0.593
Systolic heart failure	2336	233 (10.0)	142 (9.3)	91 (11.3)	0.146
Thrombophilia	2336	125 (5.4)	81 (5.3)	44 (5.4)	0.923
Total number of comorbidities	2175				<0.001
None		882 (37.8)	627 (41.0)	255 (31.6)	
One		627 (26.8)	432 (28.3)	195 (24.1)	
Two		478 (20.5)	288 (18.8)	190 (23.5)	
Three or more		349 (14.9)	181 (11.8)	168 (20.8)	
Vasoactive drugs	2336	1419 (60.7)	810 (53.0)	609 (75.4)	<0.001
Mechanical ventilation	2336	1540 (65.9)	975 (63.8)	565 (69.9)	0.003
Severe sepsis at ICU	2336	645 (27.6)	326 (21.3)	319 (39.5)	<0.001
SAPS II	2336	34.0 (26.0–44.0)	32.0 (24.0–41.0)	39.0 (31.0–49.0)	<0.001
SAPS II without age and renal points	2305	20.0 (13.0–27.0)	19.0 (13.0–27.0)	21.0 (15.0–28.0)	0.001

Categorical data are presented as an absolute number/count (percentage) and continuous data as median with IQR

*AKI* acute kidney injury, *APACHE* Acute Physiology and Chronic Health Evaluation, *ICU* intensive care unit, *non*-*op* non-operative, *post*-*op* postoperative, *SAPS* Simplified Acute Physiology Score

### Three-year outcomes

**Fig. 2:**
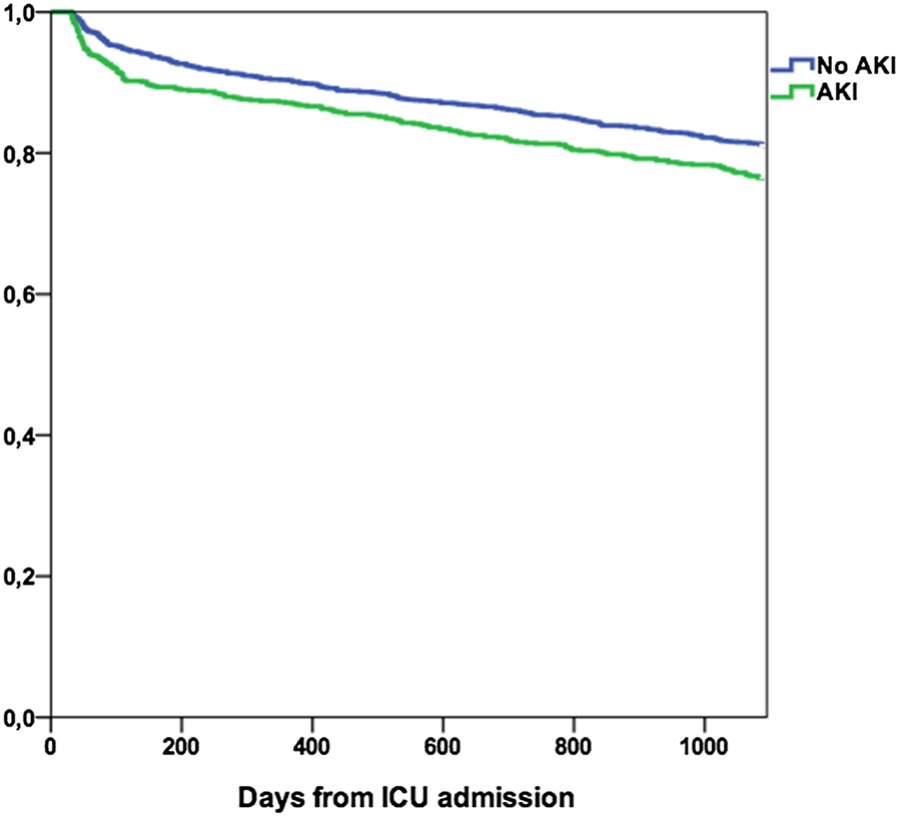
Kaplan–Meier plot presenting the cumulative survival according to the presence of acute kidney injury. *AKI* acute kidney injury

The overall 3-year mortality was 479/2336 (20.5%; 95% CI 18.7–22.1%). Crude mortality among AKI patients was higher, 190 of 808 (23.5%, 95% CI 20.6–26.4%), compared to 289 of 1528 (18.9%; 95% CI 17.0–20.9%) in the non-AKI group (*p* = 0.01). Figure 2 presents a Kaplan–Meier plot of unadjusted survival according to the presence of AKI. Mortality was 84/378 (22.2%) among stage 1, 39/162 (24.1%) among stage 2, and 67/268 (25.0%) among stage 3 AKI patients. After adjusting for age, comorbidities, and characteristics of ICU admission and treatment, AKI was not associated with an increased hazard for 3-year mortality (Table 2). Within 3 years, 20 of 808 AKI patients (2.5%; 95% CI 1.4–3.6%) and 2 of 1526 non-AKI (0.1%; 95% CI −0.1 to 0.3%) patients had developed ESRD (*p* < 0.001), with a relative risk (95% CI) of 18.9 (4.4–80.7), *p* < 0.001. Among AKI patients who commenced RRT, 19 of 192 developed ESRD (9.9%; 95% CI 5.7–14.1%).

**Table 2:** Multivariable adjusted Cox proportional hazards model for time to death during the 3-year follow-up

Characteristic	Hazard ratio (95% CI)	*p* value
Age (years)	1.03 (1.03–1.04)	<0.001
Gender (female)	0.82 (0.68–0.98)	0.047
Functional performance^a^ (normal as the reference)		
Disabled to work but no need for assistance	1.65 (1.32–2.08)	<0.001
Some assistance required	2.65 (2.00–3.54)	<0.001
Totally dependent on assistance	3.48 (2.32–5.22)	<0.001
Comorbidities^b^		
Arteriosclerosis	1.05 (0.81–1.37	0.703
Chronic kidney disease	1.53 (1.23–2.07)	0.006
Chronic obstructive pulmonary disease	1.63 (1.26–2.10)	<0.001
Diabetes mellitus	1.15 (0.92–1.43)	0.219
Hypertension	0.85 (0.69–1.04)	0.125
Chronic liver failure	2.90 (1.89–4.44)	<0.001
Malignancy	3.18 (2.18–4.62)	<0.001
Rheumatoid disease	1.87 (1.35–2.60)	<0.001
Systolic heart failure	0.96 (0.73–1.26)	0.774
Thrombophilia	0.83 (0.57–1.20)	0.313
Operative admission	1.04 (0.84–1.30)	0.710
Emergency admission	1.65 (1.16–2.33)	0.005
SAPS without age and renal points (per point)^c^	1.00 (0.99–1.01)	0.646
Vasoactive drugs	1.12 (0.90–1.39)	0.296
Severe sepsis	1.09 (0.89–1.33)	0.422
Acute kidney injury	1.05 (0.86–1.27)	0.644

Model included 2305 patients and met the assumption of constant proportional hazards

*Non*-*op* non-operative, *post*-*op* postoperative, *SAPS* Simplified Acute Physiology Score

^a^Data from five patients were missing and assumed to be normal

^b^Patients with missing data were assumed not to have the chronic condition. Number of patients with imputed data according to comorbidity: arteriosclerosis 24, chronic kidney disease 9, chronic obstructive pulmonary disease 14, diabetes 4, hypertension 14, liver failure 24, malignancy 136, rheumatoid disease 18, systolic heart failure 21, and thrombophilia 11

^c^Data missing from 31 patients

### Matched cohort

**Fig. 3:**
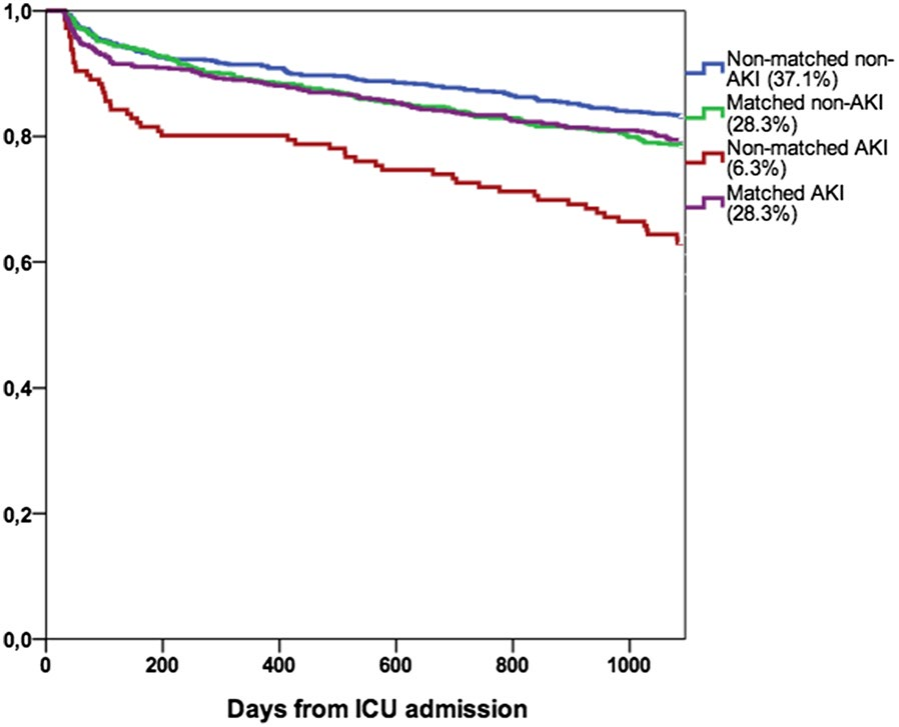
Kaplan–Meier plot presenting the cumulative survival according to matching and acute kidney injury status. The *percentage in parenthesis* presents the proportion of each group of the cohort of 2336 patients. *AKI* acute kidney injury

We found 662 matches to 662 AKI patients (81.9% of all 808 AKI patients). The groups were well balanced after matching (Table 3; Additional file 2: Figure 1). The 3-year mortality among the matched AKI patients was 136/662 (20.5%; 95% CI 17.5–23.6%) and among matched non-AKI patients 143/662 (21.6%; 95% CI 18.5–24.7%), *p* = 0.687. The absolute difference in mortality was −1.1 (95% CI −5.5 to 3.3%). The crude 3-year mortality was 54/146 (37.0%; 95% CI 29.2–44.8%) among AKI patients to whom we could not find a matched pair and 146/866 (16.9%; 95% CI 14.4–19.4%) among unmatched patients without AKI. Unmatched AKI patients had worse pre-morbid functional performance and more comorbidities than matched AKI patients (Additional file 1: Table S2). Figure 3 presents the Kaplan–Meier plot of the matched and unmatched patients according to AKI status.

**Table 3:** Characteristics of the matched patients with acute kidney injury (*N* = 662) and their propensity-matched controls (*N* = 662)

Characteristic	Matched AKI patients	Matched non-AKI patients	Standardized difference
Male gender	462/662 (69.8)	462/662 (69.8)	0
Age	63 [53–73]	63 [52–72]	1.6
Normal functional performance	332 (50.3)	342 (51.7)	−2.4
Disabled to work but no need for assistance	244 (37.0)	242 (36.6)	0.62
Some assistance required	65 (9.8)	61 (9.2)	2.0
Totally dependent on assistance	19 (2.9)	17 (2.6)	1.8
Pre-ICU risk factors for AKI			
Hypotension	151/662 (22.8)	146/622 (22.1)	1.6
Cardiogenic shock	25/662 (3.8)	21/662 (3.2)	3.2
Acute liver failure	12/662 (1.8)	12/662 (1.8)	0
Colloids	220/662 (33.2)	215/662 (32.5)	1.4
Diuretics	214/662 (32.3)	193/662 (29.2)	6.7
Angiotensin-converting-enzyme inhibitors	186/662 (28.1)	169/662 (25.5)	5.9
Radiocontrast dye	146/662 (22.1)	156/662 (23.6)	−3.5
Massive transfusion	24/662 (3.6)	20/662 (3.0)	3.4
Non-steroid anti-inflammatory drugs	60/662 (9.1)	67/662 (10.1)	−3.4
Rhabdomyolysis	20/662 (3.0)	16/662 (2.4)	3.7
Chronic kidney disease	46/658 (7.0)	32/659 (4.9)	9.0
Hypertension	349/659 (53.0)	336/659 (51.0)	3.8
Arteriosclerosis	93/657 (14.2)	88/654 (13.5)	2.0
Chronic obstructive pulmonary disease	62/660 (9.4)	70/657 (10.7)	−4.0
Heart failure	71/654 (10.9)	62/658 (9.4)	4.3
Chronic liver failure	20/658 (3.0)	15/654 (2.3)	4.4
Diabetes mellitus	160/661 (24.2)	163/660 (24.7)	2.8
Malignancy	14/619 (2.3)	18/627 (2.9)	−3.9
Rheumatoid disease	25/658 (3.8)	31/657 (4.7)	−4.5
Thrombophilia	38/661 (5.7)	39/658 (5.9)	−0.86
Emergency admission	557/662 (84.1)	560/662 (84.6)	−1.3
Operative admission	267/662 (40.3)	265/662 (40.0)	0.61
Severe sepsis	207/662 (31.3)	207/662 (31.3)	0
Mechanical ventilation	469/662 (70.8)	452/662 (68.3)	5.4
Vasoactive drugs	450/662 (68.9)	426/662 (64.4)	7.6
SAPS II without age and renal components	20.0 [14.0–27.0]	20.0 [14.0–27.0]	1.3

Categorical data are presented as an absolute number/count (percentage) and continuous data as median with IQR

*AKI* acute kidney injury, *ICU* intensive care unit, *non*-*op* non-operative, *post*-*op* postoperative, *SAPS* Simplified Acute Physiology Score

### Sensitivity analyses

After excluding patients with pre-existing CKD (N = 134), the crude 3-year mortality was 156/727 (21.5%; 95% CI 18.5–24.4%) among AKI patients and 269/1475 (18.2%; 95% CI 16.2–20.2%) among non-AKI patients (*p* = 0.075). After adjustments, AKI was not associated with an increased hazard of time to death (HR 1.02; 95% CI 0.83–1.26, *p* = 0.831) (Additional file 1: Table S3).

When AKI patients commenced RRT (*N* = 192) were compared to non-AKI patients, the crude 3-year mortality was 47/192 (24.5%; 95% CI 18.4–30.6%) compared to 288/1525 (18.9%; 95% CI 16.9–20.8%), *p* = 0.080. After adjusting for confounders, AKI necessitating RRT was not an independent risk factor for time to death during 3 years (HR 1.21; 95% CI 0.86–1.70, *p* = 0.273) (Additional file 1: Table S4).

Finally, after excluding patients with an estimated baseline Cr, pre-existing CKD, or stage 1 AKI (*N* = 1222), crude mortality among AKI patients was 68/242 (28.1%; 95% CI 22.4–33.8%) and among non-AKI patients 188/872 (21.6%; 95% CI 18.8–24.3%), *p* = 0.038. After adjusting for confounders, severe AKI was not significantly associated with time to death in 3 years (HR 1.23; 95% CI 0.91–1.65, *p* = 0.172) (Additional file 1: Table S5).

### Explanatory analyses

In the whole FINNAKI cohort (*n* = 2901) without 30-day non-survivors excluded, 252 of the 1141 (22.0%) patients with AKI had treatment restrictions (do not resuscitate, do not escalate the level of treatment, withholding of intensive care) compared to 164 of the 1596 (9.3%) patients without AKI, *p* < 0.001. When the whole cohort was included in a time-stratified model (Additional file 1: Table S6), AKI adjusted for confounders was a significant risk factor for mortality in the time period from ICU admission to 30 days, but not thereafter.

## Discussion

In this follow-up of the prospective, multicenter FINNAKI study, we found that the crude 3-year mortality among 30-day survivors was significantly higher among AKI patients compared to those without AKI. However, after adjusting for confounders known to affect long-term mortality, such as age and pre-existing comorbidities, AKI was not independently associated with decreased survival in the 3-year follow-up. The result was confirmed in a propensity-matched analysis and several sensitivity analyses.

While the role of AKI in increasing the short-term mortality is evident [3, 4, 6, 8], results regarding its role in long-term mortality are conflicting. In contrast to our findings, AKI has been found to associate with increased 1-year mortality among 30-day ICU survivors in a retrospective database analysis [5]. However, the mortality among patients without AKI was notably lower than in our study that excluded low-risk patients with uncomplicated elective post-surgical admissions, while mortality among AKI patients was corresponding, which might explain the difference between the results. Another large retrospective analysis among post-surgical hospital survivors found AKI defined by RIFLE Cr criteria to associate with decreased 10-year survival [20]. Both of these studies [5, 20] used the RIFLE Cr criterion to define AKI, and therefore, patients with oliguric AKI only were included in the control group. The long-term survival of patients with oliguric AKI without positive Cr criterion has been shown to be worse than of those without AKI but better compared to patients who fulfill both urine output and Cr criteria [21]. Therefore, the different methods of defining AKI may partly explain the controversy of these previous results to our study.

A large propensity-matched database analysis found AKI to associate with decreased survival among hospitalized patients after a median follow-up of 3 years [22]. In line with our findings, AKI was not a significant risk factor in the subgroup of critically ill patients [22]. Among cardiac surgical patients, the risk of death was higher at 1 year than at three [23], and possibly the risk of death varies over time. Corresponding to our findings, AKI treated with RRT was not associated with an increased risk of 3-year all-cause mortality among 30-day survivors compared to matched hospitalized patients without AKI [10].

A plausible explanation to our findings is that post-90-day mortality among general ICU patients is mainly determined by age and pre-existing comorbidities instead of characteristic of the acute illness [24]. In our previous sequentially matched analysis, we found the excess mortality attributable to AKI at 90 days to be 8.6% [16], which implies that AKI substantially affects the outcomes of acute illness. Since AKI and long-term mortality share the same risk factors [3, 20, 24], the higher crude 3-year mortality of AKI patients observed in the current analysis could be explained by higher age and number of comorbidities making the patients more susceptible for AKI as well as subsequent increased long-term mortality. Accordingly, instead of AKI, we found COPD, chronic liver failure, CKD, rheumatoid diseases, poor pre-morbid functional performance, and advanced age to associate with worse long-term outcome. Notably, poor pre-morbid functional status and presence of malignancy were strongly associated with decreased survival at 3 years. Our results corroborate a Scottish study that found underlying CKD among patients commenced RRT to predict long-term mortality rather than severity of acute illness [25]. Finally, our results were robust to excluding patients with pre-existing CKD, those with estimated baseline Cr, and those AKI patients who did not receive RRT.

Albeit our analysis implies that AKI is not an independent risk factor for 3-year mortality among initial 30-day survivors, it does not mean that these patients would not be in an increased risk of other adverse outcomes. An episode of AKI has been shown to be associated with elevated risk of developing CKD and ESRD [7]. Additionally, several animal models suggest that AKI can cause permanent damage on vasculature in kidney as well as outside of it [26]. Among hospitalized patients, de novo CKD developing after an episode of AKI has been shown to be an important explanatory factor for the increased long-term mortality of AKI survivors [22]. The risk of de novo CKD was found highest at three months after acute rise in Cr and to persist up to 5 years, although reducing in time [23]. The risk factors for developing de novo CKD after AKI included advanced age, pre-existing hypertension, and high Charlson comorbidity score [22], all of which are also risk factors for long-term mortality. Taken together, our analysis does not exclude the possible increase in post-3-year mortality due to potentially developing CKD and its subsequent complications such as increased cardiovascular mortality.

An obvious strength of our study was its multicenter nationwide setting, detailed prospective data collection, and a complete follow-up. Unlike other studies, with a comparably long study period including a selected cohort of patients [9, 10], our study included a heterogeneous cohort of critically ill patients, improving the external validity of our results. However, this study has some important limitations. First, inherent to all observational studies, it is impossible to adjust for unmeasured factors. For example, we had no data of pre-existing neurological conditions. We included, however, the score for pre-morbid functional performance, which reflects also aspects of neurological problems, such as dementia. Second, we did not have data about de novo CKD developing after an episode of AKI. However, the presence of CKD has been found to attenuate the mortality risk of AKI patients among hospitalized patients [22]. We found no difference between survival of AKI and non-AKI patients despite not adjusting for de novo CKD. Thus, we consider our findings representative and reliable. Third, the sample size of the matched cohort has approximately of 69% power to show a 5% difference assuming a 3-year mortality of the non-AKI group of 21.6%, and therefore, the result of no significant difference must be interpreted with caution. Additionally, despite a statistically insignificant comparison, the number of patients in different subgroups, such as those treated with RRT, is inadequate to refute a clinically significant difference in 3-year mortality due to large CI (0.86–1.70).

Finally, when interpreting our results, a potential selection bias of AKI occurring in more severely ill patients must be kept in mind. First, patients with AKI and greater illness severity more frequently had treatment restrictions, thus possibly forming the group of AKI survivors toward a healthier cohort. Second, as AKI generally occurs in the more severely ill, those who survive AKI may constitute a selected group of patients with lower risk of further adverse outcomes. Third, as encountered in other studies [10, 22], a small proportion of AKI patients (18.1%) in the current analysis was left without a match because severely ill patients without AKI could not be found. The survival curves of both the whole cohort after initial separation and the matched patients, however, are very collinear. Therefore, we consider our results to be informative in the case of corresponding baseline possibility of developing AKI and/or surviving intensive care regarding the outcome in the coming 3 years. We believe this result is of value, for example, when informing patients with AKI or their family.

## Conclusion

Among critically ill patients surviving over 30 days, AKI was not an independent risk factor for mortality at 3 years. AKI patients had higher crude 3-year mortality than non-AKI patients, but the difference was not robust for adjustments for a number of relevant confounders. Our findings imply that increased long-term mortality at 3 years among patients with AKI who survive critical illness is not related to AKI per se, but rather to advanced age and pre-existing comorbidities.

## Additional files

**Additional file 1:** **Table S1.** Logistic regression model used construct the propensity score for acute kidney injury.**Table S2.** The characteristics of all acute kidney injury patients and a comparison between matched and nonmatchedpatients with acute kidney injury. **Table S3.** The results of multivariable adjusted Cox proportionalhazards model for three-year mortality after excluding patients with chronic kidney disease. **Table S4.** Results ofmultivariable adjusted Cox proportional hazards model for time to death during the three-year follow-up amongpatients with acute kidney injury who received renal replacement therapy (n=192) and patients without acutekidney injury. **Table S5.** The results of multivariable adjusted Cox proportional hazards model for time to deathduring the three-year follow-up after excluding patients with estimated pre-admission creatinine, chronic kidneydisease, or stage 1 acute kidney injury. **Table S6.** The unadjusted and multivariable adjusted hazard ratios (HR) foracute kidney injury (AKI) in time-stratified Cox models for time to death in three-year follow-up.

**Additional file 2:** **Figure S1.** Distribution plot showing the frequency of confounders before and after matchingamong patients with and without acute kidney injury.

**Additional file 3:** **Dataset 1.** The data the paper conclusions are based on.

## Abbreviations

AIDS: acquired immune deficiency syndrome
AKI: acute kidney injury
CKD: chronic kidney disease
COPD: chronic obstructive pulmonary disease
ESRD: end-stage renal disease
FINNAKI: Finnish Acute Kidney Injury
GFR: glomerular filtration rate
ICU: intensive care unit
IQR: interquartile range
KDIGO: Kidney Disease: Improving Global Outcomes
MDRD: Modification of Diet in Renal Disease
RIFLE: risk, injury, failure, loss, end-stage renal disease
RRT: renal replacement therapy
SAPS: Simplified Acute Physiology Score
