# Supplementary material for: Three-year mortality in 30-day survivors of critical care with acute kidney injury: data from the prospective observational FINNAKI study
Source: Ann Intensive Care. 2016 Nov 29;6:118. doi: 10.1186/s13613-016-0218-5 (PMC5127925; doi:10.1186/s13613-016-0218-5)
Supplement: Supplementary file 1 — Additional file 1: Table S1. Logistic regression model used construct the propensity score for acute kidney injury.Table S2. The characteristics of all acute kidney injury patients and a comparison between matched and nonmatchedpatients with acute kidney injury. Table S3. The results of multivariable adjusted Cox proportionalhazards model for three-year mortality after excluding patients with chronic kidney disease. Table S4. Results ofmultivariable adjusted Cox proportional hazards model for time to death during the three-year follow-up amongpatients with acute kidney injury who received renal replacement therapy (n=192) and patients without acutekidney injury. Table S5. The results of multivariable adjusted Cox proportional hazards model for time to deathduring the three-year follow-up after excluding patients with estimated pre-admission creatinine, chronic kidneydisease, or stage 1 acute kidney injury. Table S6. The unadjusted and multivariable adjusted hazard ratios (HR) foracute kidney injury (AKI) in time-stratified Cox models for time to death in three-year follow-up. [file 13613_2016_218_MOESM1_ESM.pdf]

Additional File 1

Index

Table S1 2
Table S2.. 4
Table S3.. 6
Table S4.. 7
Table S5.. 9
Table S6.. 10

Table S1. Logistic regression model used construct the propensity score for acute kidney injury.

Characteristic	Odds ratio (95% confidence interval)	*p*-value
Comorbidities[a]		
Arteriosclerosis	1.22 (0.91-1.63)	0.176
Chronic kidney disease	2.43 (1.65-3.57)	< 0.001
Chronic obstructive pulmonary disease	0.85 (0.60-1.16)	0.273
Diabetes Mellitus	1.19 (0.95-1.50)	0.140
Hypertension	1.41 (1.14-1.75)	0.002
Chronic liver failure	0.98 (0.56-1.71)	0.945
Malignancy	0.85 (0.47-1.52)	0.571
Rheumatoid diseases	0.89 (0.56-1.40)	0.604
Systolic Heart failure	0.75 (0.54-1.38)	0.077
Thrombophilia	0.92 (0.62-1.38)	0.695
Premorbid Functional Performance (normal as reference)[b]		
Disabled to work but no need for assistance	1.10 (0.89-1.35)	0.384
Some assistance required	1.11 (0.80-1.55)	0.526
Totally dependent on assistance	1.46 (0.84-2.53)	0.180
Pre-ICU risk factors for acute kidney injury		
Synthetic colloids	1.34 (1.07-1.67)	0.010
Hypotension	1.63 (1-29-2.07)	< 0.001
Cardiogenic shock	1.07 (0.64-1.79)	0.797
Acute liver failure	2.03 (0.99-4.21)	0.054
Non-steroidal anti-inflammatory drugs	1.01 (0.73-1.39)	0.961
Angiotensin-converting-enzyme inhibitors	0.97 (0.76-1.24)	0.807
Radiocontrast dye	0.69 (0.55-0.86)	0.001
Rhabdomyolysis	2.84 (1.61-4.99)	< 0.001
Massive transfusion	0.86 (0.50-1.49)	0.585
Diuretics	1.40 (1.12-1.75)	0.003
Emergency admission	1.54 (1.09-2.17)	0.014

Operative admission	0.82 (0.64-1.06)	0.130
APACHE diagnosis main group	1.01 (0.99-1.04)	0.205
SAPS without age and renal points[c]	1.00 (0.99-1.01)	0.372
Mechanical ventilation during ICU stay	1.13 (0.88-1.46)	0.335
Vasoactive drugs on day 1	2.32 (1.86-2.90)	< 0.001

The model included 2335 patients. Hosmer-Lemeshow Chi-square 11.16, p=0.193.

APACHE; Acute Physiology and Chronic Health Evaluation, ICU; intensive care unit, SAPS; Simplified Acute Physiology Score.

[a]Patients with missing data were assumed not to have the chronic condition. Number of patients with imputed data according to comorbidity:

Arteriosclerosis 5, chronic kidney disease 4, chronic obstructive pulmonary disease 2, diabetes 1, hypertension 3, liver failure 4, malignancy 43, rheumatoid disease 4, systolic heart failure 8, and thrombophilia 1.

[b]Data from 2 patients were missing and assumed to be normal.

[c]Data missing from 14 patient and imputed with the median value.

Table S2. The characteristics of all acute kidney injury patients and a comparison between matched and non-matched patients with acute kidney injury.

Chracteristic	Data available	All AKI Patients N=808	Matched AKI patients N=662	Non-matched AKI patients N=146	*p*-value
Age (years)	808	64.0 (53.0-73.8)	63.0 (53.0-73.0)	66.5 (51.0-79.0)	0.026
Gender (male)	808	270 (33.4)	200 (30.2)	70 (47.9)	< 0.001
Operative admission	808	309 (38.2)	267 (40.3)	42 (28.8)	0.011
Emergency admission	808	695 (86.0)	557(84.1)	138 (94.5)	0.001
Premorbid functional performance	805				0.012
Normal		388 (48.2)	332 (50.3)	56 (38.6)	
Disabled to work but no need for assistance		303 (37.6)	244 (37.0)	59 (40.7)	
Some assistance required		85 (10.6)	65 (9.8)	20 (6.9)	
Totally dependent on assistance		29 (3.6)	8 (3.8)	21 (3.5)	
APACHE II diagnosis main groups	808				< 0.001
Respiratory		86 (10.6)	62 (9.4)	24 (16.4)	
Metabolic		63 (7.8)	53 (8.0)	10 (6.8)	
Neurologic		44 (5.4)	41 (6.2)	3 (2.1)	
Gastrointestinal, non-op		58 (7.2)	45 (6.8)	13 (8.9)	
Gastrointestinal, post-op		85 (10.5)	64 (9.7)	21 (14.4)	
Sepsis		65 (8.0)	39 (5.9)	26 (17.8)	
Renal, non-op		54 (6.7)	43 (6.5)	11 (7.5)	
Cardiovascular, non-op		89 (11.0)	76 (11.5)	13 (8.9)	
Cardiovascular, post-op		160 (19.8)	147 (22.2)	13 (8.9)	
Length of stay at ICU (days)	807	3.8 (2.1-6.7)	3.8 (2.0-6.6)	4.0 (2.5-7.0)	0.183
Length of stay at Hospital (days)		14.0 (8.0-25.0)	14.0 (8.0-24.0)	14.0 (9.0-25.0)	0.267
Acute kidney injury	808				0.121

Stage 1		378 (46.8)	317 (47.9)	61 (41.8)	
Stage 2		162 (20.0)	136 (20.5)	26 (17.8)	
Stage 3		268 (33.2)	209 (31.6)	59 (40.4)	
RRT	808	189 (23.4)	151 (22.8)	38 (26.0)	0.450
Comorbidities					
Arteriosclerosis	808	121 (15.0)	93 (14.0)	28 (19.2)	0.124
Chronic Kidney Disease	808	81 (10.0)	46 (6.9)	35 (24.0)	< 0.001
Chronic Obstructive Pulmonary Disease	808	76 (9.4)	62 (9.4)	14 (9.6)	0.877
Diabetes Mellitus	808	210 (26.0)	160 (24.2)	50 (34.2)	0.016
Hypertension	808	442 (54.7)	349 (52.7)	93 (63.7)	0.014
Chronic liver failure	808	27 (3.3)	20 (3.0)	7 (4.8)	0.306
Malignancy	757	19 (2.5)	14 (21.1)	5 (3.4)	0.363
Rheumatoid disease	808	37 (4.6)	25 (3.8)	12 (8.2)	0.028
Systolic heart failure	808	91 (11.3)	71 (10.7)	20 (13.7)	0.312
Thrombophilia	808	44 (5.4)	38 (5.7)	6 (4.1)	0.547
Total number of comorbidities	808				0.005
None		255 (31.6)	223 (33.7)	32 (21.9)	
One		195 (24.1)	163 (24.6)	32 (21.9)	
Two		190 (23.5)	151 (22.8)	39 (26.7)	
Three or more		168 (20.8)	125 (18.9)	43 (29.5)	
Vasoactive drugs	808	609 (75.4)	476 (71.9)	133 (91.1)	< 0.001
Mechanical ventilation	808	565 (69.9)	469 (70.8)	96 (65.8)	0.232
Severe Sepsis at ICU	808	319 (39.5)	207 (31.3)	112 (76.7)	< 0.001
SAPS II score	808	39.0 (31.0-49.0)	37.0 (30.0-47.0)	46.0 (35.0-54.3)	< 0.001
SAPS II score without age and renal points	808	21.0 (15.0-28.0)	21.0 (14.0-27.0)	24.0 (16.5-30.5)	0.001
Three-year mortality	808	190 (23.5)	136 (20.5)	54 (37.0)	< 0.001

APACHE; Acute Physiology and Chronic Health Evaluation, ICU; Intensive Care Unit, non-op; non-operative, post-op; post-operative, RRT; renal replacement therapy, SAPS; Simplified Acute Physiology Score. Categorical data are presented as an absolute number and percentage and continuous data as median with IQR.

Table S3. The results of multivariable adjusted Cox proportional hazards model for three-year mortality after excluding patients with chronic kidney disease.

Characteristic	Hazard ratio (CI 95%)	*p*-value
Age (years)	1.03 (1.03-1.04)	< 0.001
Gender (female)	0.87 (0.71-1.07)	0.191
Functional Performance (normal as reference)[a]		
Disabled to work but no need for assistance	1.61 (1.27-2.04)	< 0.001
Some assistance required	2.83 (2.08-3.84)	< 0.001
Totally dependent on assistance	3.27 (2.11-5.06)	< 0.001
Comorbidities[b]		
Arteriosclerosis	1.16 (0.88-1.54)	0.293
Chronic Obstructive Pulmonary Disease	1.69 (1.29-2.21)	< 0.001
Diabetes Mellitus	1.17 (0.93-1.48)	0.185
Hypertension	0.85 (0.68-1.06)	0.146
Chronic liver failure	2.55 (1.59-4.11)	< 0.001
Malignancy	3.06 (2.06-4.54)	< 0.001
Rheumatoid disease	1.79 (1.27-2.53)	0.001
Systolic Heart failure	0.95 (0.70-1.29)	0.733
Thrombophilia	0.88 (0.60-1.30)	0.521
Operative admission	1.03 (0.81-1.30)	0.812
Emergency admission	1.61 (1.10-2.34)	0.014
SAPS II without age and renal points (per point)[c]	1.00 (0.99-1.01)	0.573
Vasoactive drugs	1.14 (0.91-1.44)	0.253
Severe Sepsis	1.15 (0.92-1.43)	0.213
Acute Kidney Injury	1.02 (0.83-1.26)	0.831

SAPS; Simplified Acute Physiology Score.

Model included 2172 patients and met the assumption of constant proportional hazards.

[a] Data from 5 patients missing and assumed to be normal.

[b] Patients with missing data were assumed not to have the chronic condition. Number of patients with imputed data according to comorbidity: Arteriosclerosis 17, chronic obstructive pulmonary disease 9, diabetes 2, hypertension 10, liver failure 20, malignancy 122, rheumatoid disease 13, systolic heart failure 13, and thrombophilia 7. [c] Data missing from 30 patients.

Table S4. Results of multivariable adjusted Cox proportional hazards model for time to death during the three-year follow-up among patients with acute kidney injury who received renal replacement therapy (n=192) and patients without acute kidney injury.

Characteristic	Hazard ratio (95% confidence interval)	*p*-value
Age (years)	1.04 (1.03-1.05)	< 0.001
Gender (female)	0.80 (0.63-1.01)	0.060
Functional Performance[a] (normal as the reference)		
Disabled to work but no need for assistance	1.67 (1.28-2.19)	< 0.001
Some assistance required	2.90 (2.01-4.05)	< 0.001
Totally dependent on assistance	4.65 (2.87-7.52)	< 0.001
Comorbidities[b]		
Arteriosclerosis	1.06 (0.78-1.48)	0.646
Chronic Kidney Disease	1.63 (1.09-2.42)	0.016
Chronic Obstructive Pulmonary Disease	1.50 (1.10-2.06)	0.012
Diabetes Mellitus	1.07 (0.81-1.42)	0.636
Hypertension	0.82 (0.64-1.05)	0.113
Chronic liver failure	2.54 (1.54-4.17)	< 0.001
Malignancy	3.45 (2.19-5.46)	< 0.001
Rheumatoid disease	1.93 (1.28-2.92)	0.002
Systolic heart failure	0.81 (0.57-1.15)	0.229
Thrombophilia	0.78 (0.50-1.21)	0.266
Operative admission	1.01 (0.78-1.32)	0.923
Emergency admission	1.49 (0.98-2.27)	0.060
SAPS without age and renal points (per point)[c]	1.00 (0.99-1.02)	0.434
Vasoactive drugs	1.08 (0.84-1.39)	0.556
Severe Sepsis	1.13 (0.88-1.46)	0.333
Acute Kidney Injury	1.21 (0.86-1.70)	0.273

SAPS; Simplified Acute Physiology Score

Model included 1717 patients and met the assumption of constant proportional hazards.

[a] Data from 2 patients missing and assumed to be normal.

[b] Patients with missing data were assumed not to have the chronic condition. Number of patients with imputed data according to comorbidity:

Arteriosclerosis 17, chronic kidney disease 5, chronic obstructive pulmonary disease 9, diabetes 62, hypertension 10, liver failure 20, malignancy 110, rheumatoid disease 14, systolic heart failure 12, and thrombophilia 9.

[c] Data missing from 25 patients.

Table S5. The results of multivariable adjusted Cox proportional hazards model for time to death during the three-year follow-up after excluding patients with estimated pre-admission creatinine, chronic kidney disease, or stage 1 acute kidney injury.

Characteristic	Hazard ratio (CI 95%)	*p*-value
Age (years)	1.03 (1.02-1.04)	< 0.001
Gender (female)	0.86 (0.66-0.1.13)	0.275
Functional Performance (normal as reference)		
Disabled to work but no need for assistance	1.44 (1.05-1.96)	0.022
Some assistance required	2.72 (1.83-4.02)	< 0.001
Totally dependent on assistance	3.04 (1.76-5.26)	< 0.001
Comorbidities[a]		
Arteriosclerosis	1.36 (0.97-1.91)	0.074
Chronic Obstructive Pulmonary Disease	1.67 (1.19-2.34)	0.003
Diabetes Mellitus	1.22 (0.92-1.63)	0.171
Hypertension	0.82 (0.62-1.08)	0.147
Chronic liver failure	2.10 (1.12-3.93)	0.021
Malignancy	3.04 (1.96-4.74)	< 0.001
Rheumatoid diseases	1.66 (1.08-2.55)	0.022
Systolic heart failure	1.06 (0.75-1.50)	0.758
Thrombophilia	0.94 (0.60-1.46)	0.781
Operative admission	0.89 (0.65-1.22)	0.474
Emergency admission	1.55 (0.97-2.48)	0.068
SAPS II without age and renal points (per point)[b]	1.00 (0.99-1.02)	0.771
Vasoactive drugs	1.13 (0.84-1.51)	0.425
Severe Sepsis	1.07 (0.81-1.42)	0.622
Acute Kidney Injury	1.23 (0.91-1.65)	0.172

SAPS; Simplified Acute Physiology Score.

Model included 1098 patients and met the assumption of constant proportional hazards.

[a] Patients with missing data were assumed not to have the chronic condition. Number of patients with imputed data according to comorbidity: Arteriosclerosis 7, chronic obstructive pulmonary disease 3, diabetes 1, hypertension 2, liver failure 10, malignancy 77, rheumatoid disease 6, systolic heart failure 5, and thrombophilia 4. [b] Data missing from 1 patient.

Table S6. The unadjusted and multivariable adjusted hazard ratios (HR) for acute kidney injury (AKI) in time-stratified Cox models for time to death in three-year follow-up.

Time (days)	N	Unadjusted HR for AKI	*p*-value	N	Adjusted* HR for AKI	*p*-value
0-30	2901	1.82 [1.61-2.06]	<0.001	2858	1.41 [1.24-1.61]	<0.001
31-90	2353	1.29 [1.08-1.55]	0.006	2322	1.04 [0.86-1.27]	0.671
91-365	2222	1.17 [0.94-1.45]	0.154	2193	0.93 [0.74-1.17]	0.546
366-730	2099	1.19 [0.91-1.55]	0.198	2072	0.98 [0.74-1.31]	0.898
731-1095	1980	1.10 [0.75-1.62]	0.628	1956	0.82 [0.54-1.25]	0.354

Subjects having deceased prior to each time-period were sensored.
*Model adjusted for age, gender, pre-morbid functional performance, presence of arteriosclerosis, chronic kidney disease, chronic obstructive pulmonary disease, hypertension, chronic liver failure, malignancy, rheumatoid disease, systolic heart failure, or thrombophilia, operative admission type, emergency admission type, presence of severe sepsis in the ICU, Simplified Acute physiology score (SAPS) II points without age and renal component, and use of vasoactive drugs. If data for categorical variables were missing, the condition was assumed to be absent. Missing SAPS II scores without age and renal points were not imputed, and these patients were not included in the model.
