# Supplementary material for: Three-year mortality in 30-day survivors of critical care with acute kidney injury: data from the prospective observational FINNAKI study
Source: Ann Intensive Care. 2016 Nov 29;6:118. doi: 10.1186/s13613-016-0218-5 (PMC5127925; doi:10.1186/s13613-016-0218-5)
Supplement: Supplementary file 2 — Additional file 2: Figure S1. Distribution plot showing the frequency of confounders before and after matchingamong patients with and without acute kidney injury. [file 13613_2016_218_MOESM2_ESM.pdf]

Figure S1. Distribution plot showing the frequency of confounders before and after matching among patients with and without acute kidney injury.

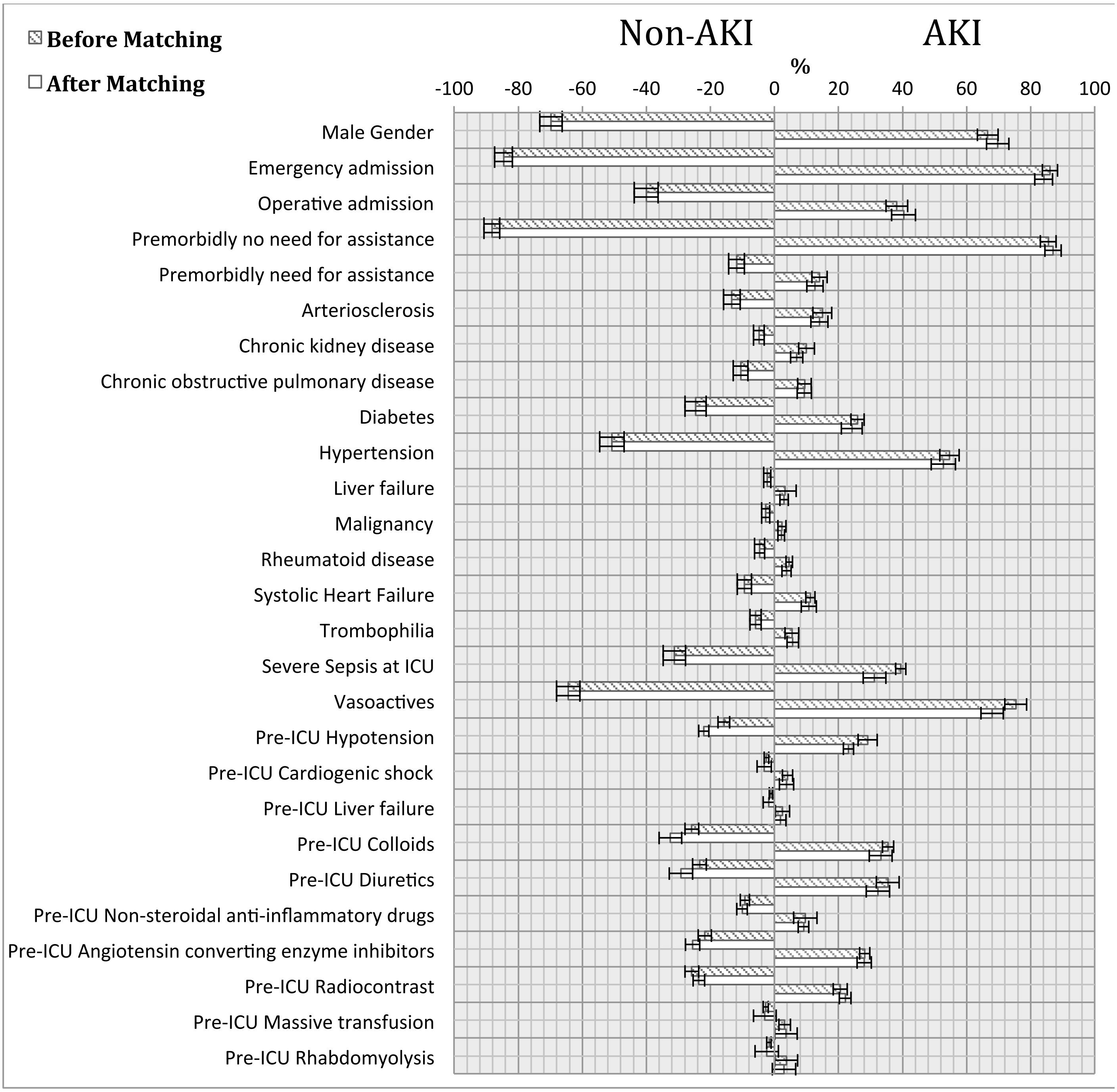

The bars show the percentage (95% confidence intervals) of patients with the characteristic.

AKI; Acute kidney injury, ICU; intensive care unit
